# Electricity in the Phenomena of Animal Life

**Published:** 1899-03

**Authors:** M. Ernest Solvay


### TEXAS MEDICAL JOURNAL.

ESTABLISHED JULY, 1885.

PUBLISHED MONTHLY.—SUBSCRIPTION $1.00 A YEAR.

VOL. XIV. AUSTIN, MARCH, 1899. No. 9.

### Selected Articles.

### Electricity in the Phenomena of Animal Life.

BY M. ERNEST SOLVAY.*

Biological research must be guided in the direction of physics and chemistry, and, in my opinion, we must set out with this profound conviction—that *the phenomena of life can and should be explained solely by the working of the physical forces which control the material universe, and that among these forces electricity plays a predominant part.*

It is with a view to contribute to the verification and development of this thesis by observation and by study of the facts of experiment that I have determined to found a special institute for research.

I would now address myself to all those who, in the future, and even after I shall have ceased to live, shall undertake researches in the laboratories which are about to be opened; I would try to state the character of the answers which I foresee are to be looked for from their labors to the great problem, as I understand it, of the nature of life. Having meditated much upon this problem, I believe that I have found some new points of view which it may be useful to make known to those who enter upon experimental researches in this direction. I aim at establishing a close correlation among the facts by tracing them back always to the foundation of physical principle. I introduce hypothesis when needful, as a lever,

*Extracts from an address delivered by M. Ernest Solvay, at Brussels, on the 14th of December, 1893, on the occasion of the public gift to the city of Brussels of the Institute for Physiological Research, founded by M. Solvay.

as a tool with which to open a new path for investigation. The future will show to what extent my views are true, or how far I have been mistaken.

### I.

### PRINCIPLES OF PHYSICOCHEMISTRY.

### I.—OF THE CONSERVATION AND TRANSFORMATION OF ENERGY—SPECIFIC CHARACTER OF ELECTRICAL ENERGY.

We know that in every case of chemical combination the quantity of heat disengaged, with positive or negative value, is equal to that which would be necessary and sufficient to decompose into its constituents the substance formed. . . . It seems permissible to assume that, in every case of chemical combination, electrical energy may exist before thermic energy, and that the latter may be but the result of a transformation of the former; that, if this way of looking at the facts is but partially sustained by experiment, the reason is to be found in our working under conditions which cause the immediate transformation, in whole or in part, of electricity into heat.

In support of this mode of looking at the facts let us notice that electricity is capable of decomposing a substance without sensibly raising its temperature, while this is far from being true in regard to heat; hence the product of an electrolysis is much greater than that of a dissociation due to heat alone. It follows that electricity appears to us as endowed with a peculiar and specific character, of which we shall have to take account. . . .

### II.—OF THE RELATIONS EXISTING BETWEEN THE PHENOMENA OF PHYSICS AND OF CHEMISTRY—UNITY OF THE PHYSICAL FORCES.

. . . We may, it seems to me, explain all phenomena, both physical and chemical, by assuming that every mass of matter (particle, molecule, or atom) is endowed with a specific character which is a mathematical function of its temperature, of its pressure, and of its potential energy or its electrical state.[1]

Expanding this proposition, here stated in its most general form, we recognize that it affords us the means of explaining, not only the attractive force acting between the parts of matter of the same kind, but also and equally well the special elective power which characterizes the chemical force called affinity; that it enables us to interpret the variations which this latter force exhibits under the influence of changes in temperature, pressure, or previous electrical state (dissociation, action in the nascent state, etc.); that in short this very function of which we are speaking must be the expression of the physical force called cohesion or of the chemical force known as affinity, according as the substances brought together are identical or different in nature.

All phenomena, physical and chemical, are thus blended, and fall into two great classes:

(1) Endothermic phenomena, which consume, absorb, or render latent a certain quantity of external energy; and

(2) Exothermic phenomena, which set free a part of the energy existing in the potential state in the ingredients which unite in the formation of a new body.

Let us go back now to what has been said (in I) as to the pre-appearance of electrical energy in the act of chemical combination.

If it be true that chemical and physical phenomena present no essential points of difference, and are but the manifestations of a single and identical aggregate of causes, have we not ground for supposing, by reason of analogy, that in every exothermic physical phenomenon the energy set free appears, or tends to appear, first under the form of electricity? That if we have not hitherto succeeded in completely revealing it in this form, it is because the very circumstances of our experiments have occasioned its transformation.

If this conjecture be vindicated we shall be authorized to regard all the phenomena of nature, whatever they may be, as manifestations of electricity, without deciding beforehand anything as to the essential nature of this force.

This is the form under which the idea presents itself to my mind of the unity of the forces of nature.

### II.

### OF THE LIVING MOTOR.

### I.—OF THE NATURE OF THE LIVING MOTOR.

. . . Among the essential characteristics presented by the animal kingdom, one of the most striking is unquestionably the faculty of automatic locomotion, which has been called motivity.

The study of this distinctive quality leads us quite naturally to the most general consideration of the phenomena of life in their connected aggregate, so that we may conveniently take this as our starting point when we propose to unfold the part taken by the forces of inanimate nature in connection with the phenomena in question. Since motivity is essentially inherent in the nature of the living animal organism, we may say that an animal is a motor; further than this, we have good reason to believe that an animal is an electric motor, as I will now try to show.

Proceeding by the method of exclusion, we see at the outset that motivity can not arise from a dynamic transformation of potential energy of the kind presented to us in a hydraulic motor, for we perceive neither a reservoir of liquid nor a fall capable of being turned to account.

Neither can the cause we seek be found in a purely thermic transformation of the same kind as that exhibited by gas engines, hot air engines, and steam engines, to which contrivances no one would think of comparing a muscle.

On the other hand, electricity lends itself to a satisfactory solution of the problem before us, a solution to which it will be well that we give our attention, at any rate until the future shall have revealed to us the existence of some other as yet unthought-of mode of transformation of energy, if so be that such a mode exist.

Laying aside, then, finally, on the evidence before us, the supposition of hydraulic action, let us examine more closely the two other hypotheses, and let us especially compare the yield in mechanical energy of a thermic or electric motor with that of a living motor.

We know that the most highly approved thermic motors do not give more than about 8½ per cent. of useful effect; the theoretic limit to the yield of steam engines is 17 per cent., and to that of gas engines 21 per cent. On the other hand, let us look back to the experiments of Hirn and Helmholtz. We find that we must assign to the yield of a muscle a mean value of more than 30 per cent.

It is impossible, as we see, to compare with this useful effect the far inferior yield of the thermic motors of human contrivance; the difference is so great that, without reference to certain physical impossibilities pointed out by Hirn, it suffices to make us give up the hypothesis of a thermic cause, in the sense in which we have just spoken of it. The idea of electrical transformation presents itself in quite a different light, since in this case the yield in dynamic effect is much greater and is comparable to that of the living motor.

### II.—OF THE SEAT IN THE ANIMAL BODY OF ELECTRICAL MANIFESTATIONS.—ELECTROGENIC APPARATUS.

. . . Oxidation of organic matter takes place in all the living cells. It takes place even in the blood and in the lymph at the expense of the chemical constituents carried forward by these fluids and not as yet endowed with organized structure. All the tissues take part in this process of internal combustion. All must thus contribute to the supply of energy necessary to the living being.

But the relative importance of each of the tissues varies greatly in this respect. To convince ourselves of this we need but, for instance, contrast the physiology of muscular tissue with that of connective tissue.

The preponderant importance of the part played by muscular tissue is manifest; we know, in fact, that when work is being done the muscles come in for some 70 or 80 per cent. of the total oxidation taking place in the animal economy. The glands, which represent apparatus composed of cells of great vital activity, also undergo rapid oxidation. Internal oxidation is for us the source of electricity. This process goes on chiefly in the muscles and in the glands; hence we give to these portions of the whole organic structure the name of electrogenic apparatus.

It is interesting to notice that the tissues which are the seat of most active oxidation, as the muscles and the glands, are richly provided with nerves, while those in which oxidation is feeble, as the cartilages and connective tissue are but poorly supplied by the nervous system.

May we not use this fact—a very important one, in my opinion—to establish an analogy between each animal organism, simple or complex, *amoeba* or man, and the cell or single element of a voltaic battery? Thus the readily oxidizable tissues, forming the electrogenic apparatus, would play the part of the negative plate, the fluids producing oxidation and hydration that of the positive plate; the nerves would serve to close the circuit.

We should thus recognize in the economy of the living animal the chemical duality necessary for the production of electrical energy; should we not, consequently, be justified in considering this organism as a true physiological battery? If so, we ought to follow up the comparison, and try to understand how this battery works; what is the nature and what the extent of its circuit; what are the causes which make its discharge to vary, and finally, what becomes of the energy which it develops.

### III.—OF THE PHYSIOLOGICAL VOLTAIC BATTERY.

In every battery the existence of available electrical energy is manifested only in so far as the electricity is able to flow off by appropriate conductors to parts of the apparatus where it may be utilized or transformed.

In default of such an arrangement—in the case, for instance, of a battery short-circuited by means of a conductor of low resistance —we perceive nothing but a development of heat, which seems to be the immediate result of the transformation of energy which occurs, but which, in accordance with what we have said as to the preappearance of electricity, may here again be regarded as a secondary phenomenon.

To justify the use of the expression physiological battery or pile, we have at the outset to prove that there exists in the animal organism lines of least electrical resistance (or behaving as such) capable of giving direction to the electricity disengaged, of conducting and distributing it.

But I have good reason for believing that this part devolves upon the nerves, and that wherever they are developed and ramified they gather up a part of the electricity produced by the electrogenic apparatus and convey it to points where it reappears unaltered or transformed.

The slight oxidizability of nervous tissue and its great functional importance lend, moreover, support to this mode of looking at it.

In what way does the propagation of electricity in the nerves take place? This question is certainly destined to occupy a large place in the program for the experimental work of the institute. To answer it there will be need, it seems to me, for seeking first of all to complete our knowledge of the constitution itself of the nerve fiber, afterwards to study very closely the electro-physical properties of nerve tissue, to investigate under what influences its electrical resistance varies, to determine finally the nature of the process itself by which the nerve current is propagated toward the organs where it is utilized. The inmost nature of this process is as yet unknown to us, and I do not understand that it is to be regarded as identical with the propagation of electricity in a metallic conductor. I limit myself then, for the present, to a single remark on this point —already proved to be true—namely, that manifestations of nerve action are always accompanied by variations of electrical condition.

I will not enlarge further upon these questions, important as they are, so as not to go beyond the plan of a merely general statement of views.

No one among you, moreover, can have overlooked the great interest of the researches of which these points will form the subject in the new institute.

IV.—OF THE PART PLAYED BY THE NERVES IN THE PHENOMENA OF STIMULATION.

Muscle and nerve respond to stimulation of mechanical, thermic, chemical, or electrical origin, but the last of these is attended with especially remarkable results.

Electrical influence is in fact by far the most powerful and extended in its effect, whether it be applied directly to the muscular tissue or, above all, if it be applied to a nerve.

On the one hand, if a muscle be directly stimulated by a shock, a drop of acid, or a prick with a sharp point, we produce but a limited local effect, while, on the contrary, if we act through the medium of a nerve we call forth generalized effects, whatever be the stimulus made use of, and it is the whole muscle that contracts as if each of its parts were electrified.

To obtain the same result without having recourse to a nerve as medium it would be necessary to apply the stimulus simultaneously to all the elementary fibers of the muscle.

These facts have a double bearing: they seem to justify the idea of attributing to the nerves the part of conductors, and show also that electricity is the form of energy which lends itself better than any other to propagation to a distance and to distribution in the living organism.

V.—OF MUSCULAR CONTRACTILITY.

Although stimulation gives rise to electrical manifestations it does not follow that the appearance or presence of electricity is necessarily dependent upon the occurrance of external stimulation.

I am much rather inclined to believe that the physiological electric battery is always in a state of activity, and that a muscle works—that is to say, gives out energy produced by this battery—even when it accomplishes no external work.

A muscle is an elastic body, more or less stretched, capable of being progressively contracted by the electric current up to the point of producing an external mechanical effect. As the current diminishes the muscle relaxes; as the current increases it contracts, and we may say that its *tonus*, or intensity of contraction, is some function of the quantity of electrical energy employed.

On the other hand, it seems evident that it is in consequence not of a static but of a dynamic action that the muscle as an organ maintains its state of contraction. It behaves like a spring upon which a jet of water constantly plays, and not like a spring loaded with a weight.

In the former of these two cases the dynamic stress, constantly kept up, is exerted against work, which involves expenditure of the moving fluid and, consequently, a certain amount of energy. In the same way the sustained contraction of a muscle also demands a continued supply of energy.

The physiological battery operates, then, to furnish this energy even when the muscle it at rest, and as no external work is accomplished in this condition all the energy furnished is transformed into heat.

It would be easy to multiply illustrations of this condition of special equilibrium; we find it notably exhibited in the case of a ball supported on a jet of water and in that of a soaring bird, which contends with the action of gravity by the rhythmic strokes of its wings.

### VI.—OF THE VARIABILITY IN YIELD OF ENERGY BY THE ANIMAL ORGANISM.

It is easy to see that the quantity of energy consumed must increase when the state of rest is followed by that of activity from whatever cause, during which external and internal work are superadded to that of the *tonus* existing during muscular repose. The reaction of the organism is not constant; it varies in intensity with the condition of sleep, of wakefulness, and of work. . . .

It must be, then,, of absolute necessity that the yield or output of energy of the physiological battery can regulate itself in due proportion, so that there shall never be either excess or deficiency.

Where shall we find the seat of this regulating action if it be not in the nervous system itself, with the importance of which as an apparatus of stimulation we have already taken note?

Assuming that the nervous system is not the producer of energy, but that its function is only to distribute it, must we not hence conclude that by means of its stimulating power it can modify, hasten or retard this distribution?

### VII.—OF THE GENERAL REASON FOR THE EXISTENCE OF THE NERVOUS SYSTEM.

We may picture to ourselves simply enough, in general principle, the reason for the existence of the nervous system if we represent a nerve as the main path by which electrical energy is carried from the point at which it originates to a nerve center, and from thence to an organ of expenditure.

In proportion as we ascend in the scale of matured animal forms or in that of embryonic stages of development, and as we study more and more perfect organisms, we observe the muscular and nervous systems develop in extent and at the same time increase in the complexity of their structure and functions. In proportion as the embryo undergoes development the nerve fibre elongates and becomes the seat of increased electrical resistance.

Thus the nervous net-work ceases to be able to carry more than a continually diminishing part of the total energy. The greater part, forced to undergo utilization at the seat of its production, gives to the muscular fiber its peculiar character and brings about its contractility or tonicity, as we have seen (in V). . . .

If we reflect upon the stage of progressive evolution of an inferior animal, or upon one of the earlier phases of development of an embryo, we understand that in proportion as nerve trunks take their rise in the neighborhood of each other differences of electric potential, existing between certain points of these trunks, tend to give rise to new trunks, superadded to the former, and the presence of which helps to render electrically dependent upon each other the different parts of the electrogenic system.

Let these *anastomoses* be multiplied *ad infinitum,* without losing sight of the fundamental idea of the system, and we shall be able, as it seems, to succeed in explaining the governmental order which reigns throughout the whole nervous system, from the smallest sporadic ganglia to the brain, which is itself the center of centers.

### VIII.—OF THE PART PLAYED BY SENSORY EXCITATIONS.

Let us now investigate how the nervous system may play the part of stimulator while drawing its functional energy from the reaction of the whole organism, and how it comes to regulate the operation in all its variations of the physiological battery.

Nothing seems to stand in the way of our looking on the series of multiplied nerve trunks which constitute the nervous system of one of the higher animals as closely analogous to an ordinary system of wires for the distribution of electrical energy, in which the whole series of apparatus for the consumption of this energy (lamps, electric motors, etc.) are branched off from a great source of production of electricity at a central station.

We may assume all of these pieces of apparatus for the utilization of the current to be regulated by instruments electrically connected with the central source of power, which instruments themselves consume in their operation a certain share of the available energy and represent so many secondary derivations of energy from the system. Finally, to complete this ideal scheme, we may imagine each of the pieces of apparatus for the utilization of the current to be furnished with an "annunciator"—an instrument operated electrically—which shall give notice to the regulating apparatus of anything going wrong at the point at which such instrument is placed.

The aggregate of all the regulating apparatus in such a system forms, to my eyes, an image of the brain, and the instruments which give notice of what may occur at the various outlying points in the system are but the analogues of the organs of sense.

Moreover, nothing prevents the parts of such a system being arranged so as to produce apparent automatism; such would be the impresssion conveyed by the innate character of the reflex actions arising from the structure conferred upon the system.

As for the sense impressions, the predominant part assigned them in such a distribution of energy will be to act upon the regulating apparatus, and by their means to modify the conditions of output of energy.

As the effect of influences from without acting upon the organism will be to oppose or to favor organic activity in those parts where such influences have long tended to favor such activity, the organs of special sense will have been formed and developed, and will have become the normal stimulators of vital action.

In such a system, provided the normal organism be not called upon for more than a part of the energy which it can produce, the actual production must essentially correspond with the demand for it; the physiological battery has, in the sensory organs, a true regulating apparatus capable of adjusting the amount of vital oxidation to the direct or indirect excitations which come from the environment; the whole matter appears as if the stimulus of sense impressions set up conditions more favorable for the genesis of energy by increasing nerve conductivity, perhaps by a swelling up and consequent increase of contact or compression of the material of the axis cylinders. The flow of energy thus produced at the seat of excitation will necessarily be returned like an echo to the other end of the line, acting upon the muscle in which energy is utilized, and the increase of muscular contraction will be the physiological expression of this echo in the muscle of the signal given by the sense impression. . . .

### III.

### THE MECHANISM OF VITALITY.

### I.—OF THE CHARACTERISTICS OF VITAL ACTIVITY.

Having thus pictured to myself the conditions of the working of the nervous system in a living animal, working which is dependent upon the distribution of electric energy generated by physicochemical reactions in the protoplasm, I have been led to examine how it is possible to conceive of the origin of vitality in this system; that is to say, the origin of the reactions producing electricity in the very heart of the primitive protoplasm.

Reasoning from one inference to another, I have come to propose in definite form the following thesis:

Life is essentially characterized by the existence of a system of continuous reactions, the necessary elements of which are ceaselessly reproduced and which take place in the midst of an appropriate medium.

It presents itself to us, therefore, not only in the complex organism, but even in the last result of its structural subdivision in the single cell, in which it still exhibits the same essential character by which it is sufficiently defined.

It is not limited by particular morphological conditions. Organic form and structure may vary almost endlessly with a condition of the medium without the essential character of vitality thereby disappearing.

In order that this vital reaction may take place three general conditions are necessary: First, the simultaneous presence together of the prime materials essential to the reaction; secondly, a particular state of distribution of these prime materials, from which may result the heterogeneity indispensable to the progress of the reaction, and, finally, such arrangements as shall permit the energy liberated by the reaction to be consumed, utilized, or transformed, either on the spot or outside the field of the reaction.

### II.—OF OXIDATION IN THE ORGANISM.

Among the chemical phenomena, varied as they are, which the living organism presents to us there is one, that of oxidation, which is distinguished from all the rest by its generality of extent and its preponderant importance; we may even say that, so far as the higher animals are concerned, oxidation is the essential chemical phenomenon of life and the others are but of secondary character. . . . The principal seat of vital activity is to be found in protoplasm submitted to the action of oxygen. It is protoplasm which forms cells; it is by it that they live and are nourished. As for the protoplasm itself, it derives its material from the various forms of food, solid, liquid, and gaseous, which are thus the prime source of the energy developed in the organism. . . . But before broaching the subject of chemical change as it occurs in the organism let us first consider a simple phenomenon, and one with which we are familiar; we shall thus make clearer the statement which is to follow.

When we plunge a piece of perfectly pure and homogeneous zinc into acidulated water, as long as the metal remains isolated in the liquid we observe no reaction, save that at the first moment of introduction a difference of electric potential is immediately established, but all is limited to this.

If, on the other hand, we connect the zinc with the acid by a substance less oxidizable than this metal, immediately an electric current is set up and reaction between zinc and acid takes place.

Simple contact then does not suffice to bring about reaction; it can but produce a sort of orientation of tension, or, if one please so to put it, a new state of equilibrium of the particles, in which there come in an electro-motive force and a counter electro-motive force equal and opposed thereto.

When the circuit is completed by means of a nonoxidizable or less oxidizable substance this equilibrium is made an end of by doing away with the counter electro-motive force, as a path of escape is offered to the electricity; but the electric flow or current thus established constitutes an available form of energy, which may be used to liberate zinc if we cause such current to act by a suitable arrangement upon an easily decomposable zinc salt.

On condition, then, that we provide by a supply of supplementary energy for the inevitable losses of effect resulting from these successive operations, we see that it is possible to form a cycle of reactions corresponding at all points to our definition of vitality.

If, then, this definition give a true statement of the facts, must we not come to recognize in the *vital cycle* the image of that which we have just sketched. . . .

Let us consider a culture medium capable of maintaining the life of a given cell by furnishing it with all the constituent material it requires, and let us observe that in order to suitability of the medium for the cell the former must contain at least the chemical elements which occur as constituents of the latter.

As long as the cell is absent this medium may be compared to the voltaic battery with circuit unclosed, which we have taken as an example by analogy. Like this latter, and by virtue of the same natural laws, it will present a state of equilibrium, due to the existence of two opposite electrical forces—a kind of state of virtual combination between the electro-positive and electro-negative constituents—but no chemical change will disturb its original homogeneity.

Let us now suppose that the living cell is introduced. As soon as equilibrium is overthrown displacements of electricity are set up, combinations and decompositions take place, and the vital circulation of energy and matter is established.

To explain what has thus occurred we must assume, it seems to me, that the first result of the introduction of the living germ or cell has been to offer to the electricity conducting paths, or in other words, to bring about what I will call *odogensis.*[1] The first effect of vitality would seem to be that of a contact action, allowing of chemical change being energetically produced by favor of the short circuits offered to the electrical energy engendered.

III.—OF THE PART PLAYED BY THE LIVING CELL.

Nevertheless, how can this take place, since the medium we have under consideration continues inactive, the cell introduced reacting only with the stratum of this medium with which it is in immediate contact?

To explain this it is essential to remark that from the quite special point of view at which we are just now placing ourselves we must logically think of the cell as chemically forming a part of the medium by which it is surrounded, since it draws from thence, and continues to draw from thence, the materials from which it is made up. The cell, although distinct from the medium, is but a fraction of it. The chemical medium thus extends to the cell which lives in it; the latter in finding its way into the former has but set up a *selection* among the constituent elements.

The fundamentally electro-positive materials go to form the substance of the cell itself; the oxygen, or its electro-negative equivalent, remains in the portion of the medium which is in intimate contact with the matter of the cell, whether on the outside or on the inside of the morphological element which it constitutes.

It follows then, clearly, from this remark that the cell is, properly speaking, but the *means of reaction* presented to the medium, and, further, that the path for electric conduction that we are looking for must exist between the cell and the fractional part of the medium which is in direct relation with it.

IV.—ON THE PART PLAYED BY SELECTIVE ELECTROLYSIS IN THE PHENOMENON OF CELL NUTRITION.

But before we concern ourselves with this point we ought to look into the phenomenon of cell nutrition and ask ourselves how it is that in the complex medium that surrounds it on all sides the cell is able in some way to choose the elementary particles which suit it; by what directive influence these particles take up their positions and arrange themselves in such a way as to become integral parts of an organized structure; whence finally comes the impulse which determines the *special course* of the vital circulation of matter and energy, allowing the cell, on the one hand, to assimilate such or such a determinate constituent from the medium, while *at the same time* it gives up, on the other hand, some constituent which has just before formed a part of its structure?

It is easy to reply to these questions if we go back to the inorganic reaction between zinc and acid, and take it as a new subject of comparison, imagining now the zinc to be in a finely divided condition. We have seen that in this case the acid, ready to combine with the zinc, is held in check by the electricity set free at the first moment of contact, this electricity counterbalancing or neutralizing its chemical affinity.

We have the same state of things before us in the growing cell surrounded by a nutrient medium. The albuminoids of the plasma correspond to the finely divided zinc; the oxygen represents the acid of the mineral illustration.

When this oxygen, kept in the inactive state by favor of the albuminoids with which it is virtually associated, makes its way with them into the domain of the cell and comes in contact with albuminoid materials of the same kind, but more readily oxidizable for reasons which we shall see further on, it at once enters into combination with them. It thus sets free new portions of albuminoid matter from the virtual combination which it had formed with them, and these immediately take advantage of the electricity produced by the oxidation to raise their own electric potential to such a point that they may be able in consequence to take their places in turn in the structural edifice of the cell thus endothermically built up. It is in this way that, quite naturally, the cycle of reactions is established which constitutes the rotation of changes in the living machine.

The mechanism which we have just explained shows how the chemical changes which represent the nutrition of the cell take place in the inmost recesses of the living substance itself, from atom to atom and from molecule to molecule, producing, in place and step by step with their occurrence, the electric energy needed for each ensuing reaction.

Following out this order of procedure, the cell may be said to withdraw from the surrounding medium the nutrient materials which are adapted to the different parts of its own substance by its proper affinity, which I will call *selective electrolytic power*, but which is merely the resultant of the previous electrical condition of the constituents concerned. The dropping out of the one constituent necessarily incites the reentrance of another precisely similar constituent. Thanks to the continuity of the rotational change going on, each particle of the cell passes in succession, all other things being equal, through the same phases of condition with that which has preceded it, from the moment of its admission to the substance of the cell up to the moment when it beomes separated from it. Before being introduced into the substance of the cell the atom must leave the *virtual combination* in which it was held. It is precisely the disappearance of the atom which precedes that furnishes to the one which follows it the exact amount of energy required to effect its *selective electrolysis* or its liberation from the state of virtual combination. Thus we shall have concordance, referring to the possibilities of the case, between what the one lacks in order to leave the medium and enter into the substance of the cell and that which the other provides when it frees itself from the combination in which it has been held in order to engage itself in one more powerful; and, on the whole, quantitatively speaking, there will be a certain available excess of energy on the latter side. It follows from this that the points of accretion, howsoever distributed as to position in the protoplasm, always retain their identity both in composition and structure; and, as the same causes everywhere bring about the same effects, the whole cell must reproduce itself indefinitely with maintenance of its identity if the nutrient medium remain on its part constant in character and the process of transformation go on. . . .

The preceding hasty sketch sufficiently shows, I think, that it is legitimate to compare, as I have done at the outset, the mechanism of vital activity to that of a single element of a voltaic battery, when once we recognize in the living cell the special part which I have assumed it to play. . . .

I believe that these general considerations will suffice to render intelligible, and in a provisional way to justify, the mode in which

I conceive of the mechanism of life, with the aid of the physical and chemical forces of nature only, and without invoking any mysterious or extra-natural cause. . . .

[The remainder of M. Solvay's discourse is devoted to an examination of the phenomena of animal growth, reproduction, evolution (modification of species), and physical activity, in the light of the views which he holds and advocates. But it is not possible to present this latter part of the discourse in intelligible abstract within the limits assigned, and reference must be made to the original in its complete form.

The author concludes by saying: "I await now, as the result of the most rigorous course of experiment pursued in the broadest and most extensive manner, an impartial and thorough examination of the scheme which has been the subject of much of my thought for so many years."

Any one interested in the line of thought and of research suggested by this discourse would do well to address a request for a copy of the original discourse *in extenso* to M. Ernest Solvay, senateur, 43 Rue des Champs Elysees, Bruxelles, Belgium.—J. W. Mallet, in *Smithsonian Reports.*

